# The Population Genetics of Cultivation: Domestication of a Traditional Chinese Medicine, *Scrophularia ningpoensis* Hemsl. (Scrophulariaceae)

**DOI:** 10.1371/journal.pone.0105064

**Published:** 2014-08-26

**Authors:** Chuan Chen, Pan Li, Rui-Hong Wang, Barbara A. Schaal, Cheng-Xin Fu

**Affiliations:** 1 The Key Laboratory of Conservation Biology for Endangered Wildlife of the Ministry of Education, College of Life Sciences, Zhejiang University, Hangzhou, China; 2 Hangzhou Botanical Garden, Hangzhou, China; 3 Department of Biology, Washington University, St. Louis, Missouri, United States of America; East China Normal University, China

## Abstract

**Background:**

Domestic cultivation of medicinal plants is an important strategy for protecting these species from over harvesting. Some species of medicinal plants have been brought into cultivation for more than hundreds years. Concerns about severe loss of genetic diversity and sustainable cultivation can potentially limit future use of these valuable plants. Genetic studies with comprehensive sampling of multiple medicinal species by molecular markers will allow for assessment and management of these species. Here we examine the population genetic consequences of cultivation and domestication in *Scrophularia ningpoensis* Hemsl. We used chloroplast DNA and genomic AFLP markers to clarify not only the effects of domestication on genetic diversity, but also determine the geographic origins of cultivars and their genetic divergence from native populations. These results will allow both better management of cultivated populations, but also provide insights for crop improvement.

**Results:**

Twenty-one cpDNA haplotypes of *S. ningpoensis* were identified. Wild populations contain all haplotypes, whereas only three haplotypes were found in cultivated populations with wild populations having twice the haplotype diversity of cultivated populations. Genetic differentiation between cultivated populations and wild populations was significant. Genomic AFLP markers revealed similar genetic diversity patterns. Furthermore, Structure analysis grouped all wild populations into two gene pools; two of which shared the same gene pool with cultivated *S. ningpoensis*. The result of Neighbor-Joining analysis was consistent with the structure analysis. In principal coordinate analysis, three cultivated populations from Zhejiang Province grouped together and were separated from other cultivated populations.

**Conclusions:**

These results suggest that cultivated *S. ningpoensis* has experienced dramatic loss of genetic diversity under anthropogenic influence. We postulate that strong artificial selection for medicinal quality has resulted in genetic differentiation between cultivated and wild populations. Furthermore, it appears that wild populations in Jiangxi-Hunan area were involved in the origin of cultivated *S. ningpoensis*.

## Introduction

Plant domestication is one of the great milestones in human history. The process of domestication represents a continuum of increasing codependence between plants and people [1]. Crop species have been derived from their wild progenitors as a result of artificial selection for desirable traits by early farmers. In the light of crop domestication, a number of agencies now are recommending that wild medicinal plants be brought into cultivation systems [2], [3]. Currently, more than 80% of the world's population in developing countries depends primarily on herbal medicine for basic healthcare needs [4], [5]. Given the demand for a reliable and uniform supply of medicinal plants and the accelerating depletion of forest resources, cultivation of medicinal plants species would be an important strategy and a viable alternative to harvesting of natural populations [6], [7].

At present some medicinal plants are grown in home farms, and some are cultivated as field crops. But the number of medicinal plants species currently in high intensity cultivation for commercial production are varied world-wide. In India, 20 species are currently under cultivation, in Hungary 40 and in Germany 100 [6], [8], [9], [10]. China, with its history of thousands years of medicinal plant use cultivates only about 250 species [11], [12]. Considering the harvest pressure on wild populations and the increasing demands, the most popular medicinal plants are cultivated under the supervision of Chinese government on large scale reaching almost 1 million acres [13].

As the demand for medicinal plant cultivation increased, many studies examined the impact of cultivation on genetic diversity, species such as *Artemisia judaica* [14], *Scutellaria baicalensis* [15], *Corydalis yanhusuo* [16], *Coptis chinensis* [17], *Magnolia officinalis* subsp. *biloba* [18], *Fritillaria cirrhosa* [19]. However, all these studies only used single molecular marker or local sampling which just revealed genetic diversity patterns in limited populations. Since genetic diversity underlies the plasticity of secondary metabolism and hence the production of medicinal compounds [20], understanding the population genetics across the range of wild populations is essential for utilizing native resources for improvement of cultivated species. A study with comprehensive sampling of both cultivated and wild populations of medicinal species, using both chloroplast and nuclear DNA markers, can clarify not only changes in genetic diversity, but also provide information on domestication including the geographic origins, ancestor populations and the impact of domestication on population genetics.

Plant secondary metabolites are responsible for phytochemical diversity and therapeutic efficacy of medicinal species. The production of secondary compounds is influenced by both environmental and genetic factors [20], [21]. Thus geographically distinct populations even of the same species may have vastly different medical qualities [22]. Modern methods of phytochemical methods are used to discriminate geographically dispersed cultivars within a medicinal species [23], [24], [25], [26], [27]. Yet little is known about the genetic differences among the wild populations of a medicinal species and whether cultivated populations with high pharmaceutical quality have already significantly diverged from wild populations. Additionally, understanding genetic changes associated with cultivation not only helps control the quality of medicinal herbs, but also provides practical information essential for formulating appropriate conservation and cultivation management strategies [28], [29].

*Scrophularia ningpoensis* Hemsl. is a perennial herb native to Southeastern China. Wild populations are distributed in forests along streams, thickets, and tall grasses below alt. 1700 m and reproduce from seed [30]. Roots of this medicinal herb have a long history of widespread use in China to treat inflammation, laryngitis, tonsillitis, abscesses and constipation [31], [32], [33]. The first recorded use in ancient Chinese literature is from ca. 100 BC [34]. Harpagoside, angroside C, acteoside and cinnamic acid are the main bioactive components [35], [36], [37], [38], [39]. According to the Chinese Pharmacopoeia, only the root of *S. ningpoensis* is listed as the Radix Scrophulariae [40]. Our recent field investigations indicated that wild populations of this herb have suffered rapid declines, and the species is extirpated in many locations due to over exploitation and deterioration of habitats. An analysis of the population genetics of wild *S. ningpoensis* populations is important and urgent for appropriate utilization, conservation and preservation of the wild resources.

Today, *S. ningpoensis* is cultivated for its roots on a large scale in several regions of Central and Southeastern China. This species can trace its cultivation history back to Song dynasty (1000 years ago) beginning in backyard gardens [41]. In spite of this long written history, the geographic origin of cultivation has not been recorded. Formal cultivation programs were initiated in China only since the 1950's [42]. Unlike wild populations, cultivated *S. ningpoensis* is propagated vegetatively from rhizome. During the cultivation, large rhizomes are often selected for propagation; flowers are removed before opening so that resources are allocated to vegetative growth, in particular the rhizomes [43], [44]. Our previous study used HPLC revealed that among cultivated *S. ningpoensis*, accessions from Zhejiang Province produced higher concentrations of three main bioactive compounds, suggesting that the best quality of cultivated *S. ningpoensis* is from Zhejiang [45]. These results are consistent with the general perspective that *S. ningpoensis* from Zhejiang has the best quality for medical use [46]. Besides the phytochemical differences, the unresolved question of whether cultivated *S. ningpoensis* from Zhejiang is genetically differentiated for others is also important and indispensable to sustainable cultivation of *S. ninpoensis*.

Here we used two chloroplast DNA fragments and AFLP markers to study the population genetics and phylogeography of wild and cultivated *S. ningpoensis* with the aims of: (1) evaluating the genetic erosion of cultivated populations and the change in the pattern of genetic diversity under artificial selection; (2) unraveling the geographical origins and ancestral populations of cultivated *S. ningpoensis*; (3) clarifying the genetic divergence between cultivated populations from Zhejiang Province and the rest of cultivated *S. ningpoensis*; and (4) determining which wild populations are valuable and can be introduced for improvement of cultivation.

## Materials and Methods

### Ethics Statement

Management Bureau of Mt. Jinggang National Nature Reserve issued the permit for Jinggang Mountain (JGW); Management Bureau of Mt. Tianmu National Nature Reserve issued the permit for Tianmu Mountain (TNW, TM1W, TM2W); Management Bureau of Mt. Dapan National Nature Reserve issued the permit for Dapan Mountain (TWW). No specific permissions were required for other locations which are neither privately owned nor protected and the field study did not involve endangered or protected species.

### Sampling and DNA extraction

**Figure 1:**
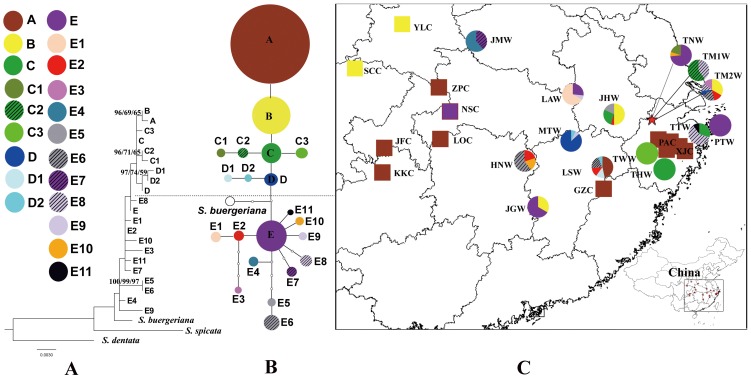
Analyses of 21 chloroplast (cp) DNA haplotypes of *Scrophularia ningpoensis*. (A) The maximum likelihood tree of 21 chloroplast (cp) DNA haplotypes of *S. ningpoensis*, *S. buergeriana*, *S. spicata* and *S. dentata* were used as the outgroup. Statistical supports (Bayesian posterior probability ≥80/maximum likelihood bootstrap value ≥50/maximum parsimony bootstrap value ≥50) are indicated on the branches. (B) 95% plausible network of the 21 cpDNA haplotypes of *S. ningpoensis* (A–E11). The size of circles corresponds to the frequency of each haplotype. Each solid line represents one mutational step that interconnects two haplotypes for which parsimony is supported at the 95% level. The small open circles indicate inferred intermediate haplotypes not detected in this investigation. (C) A geographic distribution of 21 cpDNA haplotypes (A–E11) detected in *Scrophularia ningpoensis*. The populations correspond to those detailed in Table 1. Squares represent cultivated populations and circles represent wild populations.

Field studies were conducted throughout distribution range of *Scrophularia ningpoensis*. Specific locations (GPS coordinates) of our field work are indicated in Table 1. A total of 364 individuals representing 13 cultivated and 14 wild populations were sampled with nine to twenty-two individuals collected randomly for each population (Table 1, Figure 1). Leaf material was dried in silica gel and stored at 4°C. Voucher specimens are deposited in the Herbarium of Zhejiang University (HZU), China. Total genomic DNA was extracted using a modified (CTAB) method [47], [48].

**Table 1:** Details of sample locations and sample sizes (N) of *Scrophularia ningpoensis*.

Population code	Locality	Altitude (m)	Lat. (°N) Long. (°E)	N CpDNA	N AFLP	Vouchers
**CULTIVATED**						
GZC	Guangze, Fujian Province	234	117°20′, 27°32′	15	15	LP0805GZ
JFC	Jinfo Mountain, Chongqin Province	1282	107°12′, 29°00′	14	13	FU0810321
SCC	Guangyuan, Sichuan Province	594	105°47′, 32°27′	14	19	GW20070705
XJC	Xianju, Zhejiang Province	63	120°44′, 28°51′	10	10	CC20060601
*RCC/PAC	Pan'an, Zhejiang Province	356	120°24′, 28°54′	15	4	CC20060602
*YCC/PAC	Pan'an, Zhejiang Province	357	120°33′, 29°05′	11	4	CC20060603
*SHC/PAC	Pan'an, Zhejiang Province	537	120°39′, 29°08′	10	4	CC20060604
*DPC/PAC	Pan'an, Zhejiang Province	581	120°32′, 28°59′	14	4	CC20060605
YLC	Yangling, Shanxi Province	459	108°04′, 34°15′	15	10	LP20080811
ZPC	Zhenping, Shanxi Province	1013	109°33′, 31°45′	15	10	LP20090610
KKC	Suiyan, Guizhou Province	1572	107°09′, 28°13′	10	8	WY20090618
LOC	Long Mountain, Hunan Province	843	109°43′, 29°36′	15	10	ST20090601
NSC	Enshi, Hubei Province	1606	110°12′, 30°44′	13	10	ZZ20080721
**WILD**						
HNW	Pingjiang, Hunan Province	1428	113°49′, 28°38′	15	15	SS20070821
JGW	Jinggang Mountain, Jiangxi Province	975	114°07′, 26°35′	15	15	CC0708J029
JHW	Jiuhua Mountain, Anhui Province	1246	117°49′, 30°28′	22	22	CC20080507
JMW	Junma, Henan Province	673	111°25′, 33°28′	13	13	SS20070703
LAW	Tiantang zhai, Anhui Province	492	115°47′, 31°11′	15	14	LP0906508
LSW	Lenshui, Jiangxi Province	80	117°11′, 28°15′	15	15	SS20070701
MTW	Matou, Jiangxi Province	101	115°53′, 29°27′	15	15	SS20070702
PTW	Putuo, Zhejiang Province	17	122°23′, 30°00′	9	9	CC20080527
THW	Tiantai Mountain, Zhejiang Province	960	121°05′, 29°14′	15	14	LP0910919
TNW	Tianmu Mountain, Zhejiang Province	1477	119°25′, 30°20′	15	14	LP0906TMS
TTW	Tiantong, Ningbo, Zhejiang	348	121°47′, 29°48′	10	10	CC20071001
TM1W	Tianmu Mountain, Zhejiang Province	315	119°26′, 30°19′	10	10	CC20061001
TM2W	Tianmu Mountain, Zhejiang Province	266	119°29′, 30°19′	15	10	CC20070706
TWW	Dapan Mountain, Zhejiang University	469	120°23′, 28°54′	9	9	CC20060901

*In AFLP analysis, four populations RCC, YCC, SHC and DPC are combined as one population coded PAC, and in Figure 1C these four populations are also indicated as PAC.

### Chloroplast (cp) DNA sequences analysis

After preliminary screening for variation in the intergenic spacer (IGS) regions of chloroplast DNA, two regions: *trn*L–*trn*F [49] and *psb*A–*trn*H [50] were chosen for analysis because they contained sufficient levels of informative polymorphic sites. PCR amplification was performed in an ABI 9700 thermocycler (Applied Biosystems). Cycling conditions were 95°C for 2 min followed by 35 cycles of 94°C (45 s), 57°C (45 s), 72°C (1 min), and concluding extension at 72°C (10 min). The reaction mixture (25 µL) contained 2.5 mm/L MgCl_2_, 0.5 µm/L dNTP, 2.5 µl 10×buffer, 2.5 µm/L primer, 1 U Taq DNA polymerase (*Takara* Bio Inc.) and 20 ng DNA template. PCR products were identified on 1.5% agarose gels in a 0.5×TBE buffer, stained with ethidium bromide (0.5 µg/ml), visualized with ultraviolet light and photographed. DNA Marker DL2000 (*Takara* Bio Inc.) was used. The PCR products with a single band were cleaned by a GENECLEAN II Kit (BIO 101, Inc. Carlsbad, USA) for direct sequencing. Samples were sequenced in forward and reverse directions using the dideoxy chain termination method with fluorescent labeling with Big Dye Terminator (version 1.1). Sequences were determined with an ABI 377XL DNA sequencer and edited using Sequencher version 4.0 (Gene Codes Corp., Ann Arbor, MI, USA). Sequences of the two IGS regions were combined and aligned by Clustal W version 1.8 [51].

Sequences from all individuals were characterized for their cpDNA haplotypes. Chloroplast DNA haplotype diversity (*h*) and nucleotide diversity (*π*) [52] were calculated for each population (*h*_S_, *π*_S_) and overall (*h*_T_, *π*_T_) using DNASP 5.10 [53]. A haplotype network rooted by *Scrophularia buergeriana* that contains all linkages with >95% probability of being most parsimonious was drawn by TCS version 1.21 [54]. In this analysis, indels and one inversion were treated as single mutation events. Phylogenetic relationships among cpDNA haplotypes of *S. nignpoensis* were assessed by Bayesian inference (BI), maximum likelihood (ML), and maximum parsimony (MP) methods with S. *buergeriana*, *S. spicata* and *S. dentate* as the outgroup. For Bayesian inference, the substitution model was determined by MrModeltest 2.2 [55] and the best-fit model (F81+I+G) was selected by Akaike information criterion (AIC). Then Bayesian analysis was performed by MrBayes 3.1.2 [56]. Two independent runs of four Markov chains each starting with a random tree were processed in ten threads simultaneously for 50 million generations, sampling trees at every 1000 generation. The first 12500 sampled trees (25%) were discarded as burn-in samples. Maximum likelihood [57] tree and ML bootstrap searches were conducted using RAxML V.7.2.8 [58] on the CIPRES [59]. Final tree was evaluated and optimized under GAMMAGAMMA Model. ML bootstrap values were estimated from 1000 bootstrap replicates. Maximum parsimony analyses were conducted using PAUP* version 4.0b10 [60]. Support values for the relationships were calculated by performing bootstrap analyses of 1000 heuristic search replicates using the TBR branching swapping algorithm with 100 random additions per replicate. The geographical distribution of haplotypes was plotted on a map of China using Mapinfo (Pitney Bowes, Inc.). To test for significant difference in haplotype frequencies between wild and cultivated populations, total diversity (*h*_T_), within-population diversity (*h*_S_), U test (Nst/Gst) and population differentiation (*G*_ST_) were calculated in HAPLONST [61]. Hierarchical structure of genetic variation was estimated by an analysis of molecular variance (AMOVA) on three levels: among groups (cultivated and wild groups); among-population within groups; and within-population. All AMOVAs were performed with ARLEQUIN [62]. Sequence variation was tested for deviations from neutrality by Tajima's *D* statistic [63], and by Fu and Li's D* and F* statistics [64] using DNASP 5.10 [65].

### AFLP analysis

AFLP method followed the protocol established by Vos [66] with minor modifications. First, selective primer pairs were pre-screened for three individuals. Second, thirty-six primer combinations which had produced well-separated fragments in the pre-screen were used on eight individuals from different populations to determine polymorphisms. Six primer pairs which yielded polymorphic, clear and reproducible fragments were selected for the final AFLP analysis: *Eco*RI-AAC (FAM)/*Mse*I-CTC, *Eco*RI-ACA (FAM)/*Mse*I-CTG, *Eco*RI-ACC (HEX)/*Mse*I-CAC, *Eco*RI-ACC (HEX)/*Mse*I-CTA, *Eco*RI-AGG (TAMAR)/*Mse*I-CAG and *Eco*RI-AGG (TAMAR)/*Mse*I-CTC. FAM, HEX and TAMAR are three differential fluorescence labels for multiplexed electrophoresis.

The protocols are as follows: (i) in the digestion step, 2.5 ul (0.25–0.3 ug) of genomic DNA was restricted in 2.5 ul of a reaction mixture that contained 0.2 µl Eco R I (15 U/µL), 0.15 ul Mse I (10 U/µL), 0.5 µl 10×Buffer and 1.65 µl ddH_2_O. The mixture was kept at 37°C for 3 hours, then 65°C for 10 minutes; (ii) the ligation reaction included 5.0 µl digested genomic DNA, 1.0 µl 10× T4 DNA ligase buffer, 1.0 µl Eco R I-adapters (5 µM), 1.0 µl Mse I adapters (50 µM), 0.1 µl T4 DNA ligase (3 U/µL) and 1.9 µl ddH_2_O. The ligation reaction was 16°C for 12 h and the product was diluted with 90 µl of ddH_2_O; (iii) pre-selective amplifications were carried out in 12.5 µl reactions: 2.5 µl diluted ligation product, 1.25 µl 10×PCR buffer, 1 µl MgCl_2_ (25 mmol/L), 2 µl dNTPs (2.5 mmol/L), 0.5 µl Eco R I - A primer (5 umol/L), 0.5 µl Mse I - C primer (5 umol/L), 0.05 µl Taq DNA Polymerase (5 U/µL), 4.7 µl ddH_2_O. PCR conditions for pre-selective amplification were 72°C for 2 min; 94°C for 20 sec, 56°C for 30 sec, 72°C for 2 min, for 20 cycles, 60°C for 30 sec. The product of the pre-selective PCR was diluted with 150 µl ddH_2_O; (iv) selective amplification reactions included 3 µl diluted pre-selective amplification products, 1.25 µl 10×PCR buffer, 1.25 µl MgCl_2_ (25 mmol/L), 2 µl dNTPs (2.5 mmol/L), 0.8 µl Eco R I primer (0.5 U/µL), 0.5 µl M primer (5 U/µL), 0.5 µl BSA (20 mg/mL), 0.1 µl Taq DNA Polymerase (5 U/µL), 3.6 µl ddH_2_O. PCR conductions were 94°C for 2 min; 13 cycles of 94°C for 30 sec, 65°C–56°C (−0.7°C/cycles) for 1 min, 72°C for 1.5 min; 23 cycles of 94°C for 30 sec, 56°C for 1 min, 72°C for 1.5 min; 72°C for 8 min. The product of the selective amplification was analyzed on ABI3730 (Applied Biosystems) according to the manufacture instructions with GS ROX-labelled 500 size standard (Applied Biosystems). For each primer combination, negative amplification controls without template DNA resulted in no amplification products.

Raw AFLP data were scored and collected as presence/absence matrix firstly by Genemarker v1.7 (SoftGenetics LLC) and then each locus was manually inspected. Only unambiguously detectable fragments in the size range 75–450 bp were scored. To test the reproducibility of the AFLP data, the complete AFLP procedure (digestion, ligation, pre-selective, selective amplification) was repeated for 15 individuals (5% of the samples) and scored independently for each primer pair.

Assuming a set of measures of wild and cultivated populations, population genetic statistics were generated by the program POPGENE version 1.31 [68] including: the number of polymorphic fragments (*F*p), the percentage of polymorphic fragments (*PPF*), Nei's [67] gene diversity (*h*), Shannon's diversity index (*H*), coefficient of gene differentiation (*G*_ST_ = total genetic diversity - gene diversity within population/total genetic diversity), and the level of gene flow: *N*_m_ = 0.5(1−*G*_ST_)/*G*_ST_. To explore the relationships among individuals Principal Coordinate Analysis (PCoA) was performed by software MVSP version 3.1 (Kovach Computing Services, Anglesey, Wales) from pairwise Euclidian distances between individuals genotypes. A neighbor-joining (NJ) tree rooted by the populations of *Scrophularia buergeriana* was constructed by the PHYLIP 3.62 package [69] and group support was assessed by a bootstrap analysis with 1000 replicates. Genetic admixture analysis was implemented in STRUCTURE version 2.2.3 [70] with “no admixture model” and assumed “uncorrelated allele frequencies” [71]. The main modeling assumptions of STRUCTURE are Hardy-Weinberg equilibrium within populations [70]. But the cultivated populations are Hardy-Weinberg disequilibrium because of extensively clonal reproduction by rhizome within population. So instead of using all cultivated populations, only one individual from each different cultivated population (total ten individuals) were used for STRUCTURE analysis. The number of clusters (K) was set from 2 to 14 (number of all wild populations) with 10 replicate runs for each K, a burn-in period of 100000, and additional 1000000 replicates of the MCMC chains after burn-in. The structure computations output files were carried out by the freely accessible STRUCTURE HARVESTER [72] to calculate similarity coefficients between the replicate runs and to plot the mean logarithmic likelihood of K values (the means of the estimated log posterior probability of the data over the replicate runs for each K value). All populations were divided into two groups (cultivated and wild) and analysis of molecular variance (AMOVA) [73] was conducted by ARLEQUIN [62] to quantify genetic differentiation at different hierarchical levels. “Frequency- down-weighted marker values” (DW) [74] quantified by the amount of particular AFLP markers in the total dataset were calculated by AFLPdat [75].

## Results

### CpDNA sequence data

Total 21 different cpDNA haplotypes (A-U) were identified among the 364 individuals from 27 populations of *S. ningpoensis*. A total of 26 polymorphic sites were detected across the two chloroplast regions (Table 2); 21 were single-site mutations, four were length polymorphisms (4 bp, 9 bp, 7 bp and 2 bp, respectively) and one was an inversion (22 bp). Considering that sequencing poly-N regions could easily cause homoplasies due to polymerase error, length variations in mononucleotide repeats (poly A or T stretches) were not treated as polymorphisms. The *trn*H-*psb*A region (21 polymorphic sites detected in 466 aligned positions; 4.5%) was more variable than the *trn*L-*trn*F region (5 polymorphic sites detected in 811 aligned positions; 0.62%). The combined cpDNA sequences of *S. ningpoensis* varied in length from 1255 to 1268 bp, with a consensus length of 1277 bp after alignment. When the two fragments were combined, 2.0% of all sites (26/1277) were polymorphic. Both Tajima's test (*psb*A-*trn*H: *D* = −0.42908, *P*>0.10; *trn*L-*trn*F: *D* = 0.03414, *P*>0.10) and Fu & Li's test (*psb*A-*trn*H: D* = 1.26305, *P*>0.10; F* = 0.68518, *P*>0.10; *trn*L-*trn*F: D* = 1.01550, *P*>0.10; F* = 0.80528, *P*>0.10) indicated no significant deviation from neutrality for the two regions. The combined sequences of the spacers conformed to the expectation of neutrality as well (*D* = −0.33903, *P*>0.10; D* = 1.51081, 0.10>*P*>0.05; F* = 0.87503, *P*>0.10). Sequences of the eighteen *psb*A-*trn*H and six *trn*L-*trn*F haplotypes are available in GenBank database (GenBank accession numbers:KJ194143-KJ194166).

**Table 2:** Chloroplast DNA sequence polymorphisms detected in two intergenic spacer (IGS) regions of *Scrophularia ningpoensis* identifying 21 haplotypes (A-E11).

Halpotype	Nucleotide position																									
	*psb*A*-trn*H																					*trn*L*-trn*F				
	5	6	8	1	2	5	7	9	9	1	1	1	1	1	2	2	2	2	3	3	4	8	1	1	2	5
				2	1	3	4	1	2	2	4	5	6	8	4	7	7	8	5	8	0	1	1	6	1	5
										0	5	5	0	3	9	0	6	7	5	0	9		3	2	9	5
A	A	T	1^a^	C	0	0	C	A	C	0	A	T	C	T	G	T	A	T	A	2^f^	T	T	C	C	C	A
B	.	.	.	.	.	.	.	.	T	.	.	.	.	.	.	.	.	.	.	.	.	.	.	.	.	.
C	.	.	.	.	.	.	.	.	T	.	.	.	A	.	.	.	.	.	.	.		.	.	.	.	.
D	.	.	.	.	.	.	.	.	T	.	.	.	A	.	.	.	.	.	.	.	.	.	.	.	A	.
E	.	.	.	.	.	.	.	.	T	.	.	.	A	.	.	A	T	.	.	.	.	.	.	.	A	.
C1	.	.	.	.	.	.	.	.	T	.	.	.	A	G	.	.	.	C	.	.	.	.	.	.	.	.
C2	.	.	.	.	.	.	.	.	T	.	.	.	A	G	.	.	.	.	.	.	.	.	.	.	.	.
C3	.	.	.	.	.	.	.	.	T	.	.	G	A	.	.	.	.	.	.	.	.	.	.	.	.	.
D1	.	.	.	.	.	.	.	.	T	.	.	.	A	.	A	.	.	.	.	.	.	.	.	.	A	C
D2	.	.	.	.	.	.	.	.	T	.	.	.	A	.	A	.	.	.	.	.	.	.	.	.	A	.
E1	.	.	.	.	.	.	.	.	T	.	.	.	A	.	.	A	T	.	C	.	.	.	.	.	.	.
E2	.	.	.	.	.	.	.	.	T	.	.	.	A	.	.	A	T	.	.	.	.	.	.	.	.	.
E3	.	.	.	.	.	1^d^	.	.	T	.	.	.	A	.	.	A	T	.	.	2^g^	G	C	.	.	.	.
E4	T	.	0	T	.	.	.	.	T	.	.	.	A	.	.	A	T	.	.	.	.	.	.	.	A	.
E5	.	.	.	.	.	.	.	G	T	.	.	.	A	.	.	A	T	.	.	.	G	.	.	A	A	C
E6	.	.	.	.	.	.	.	G	T	1^e^	.	.	A	.	.	A	T	.	.	.	G	.	.	A	A	C
E7	.	.	.	.	.	.	T	.	T	.	.	.	A	.	.	A	T	.	.	.	G	.	.	.	A	.
E8	.	.	1^b^	.	.	.	.	.	T	.	.	.	A	.	.	A	T	.	.	.	.	.	T	.	A	.
E9	.	.	.	.	.	.	.	.	T	.	C	.	A	.	.	A	T	.	.	.	.	.	.	.	A	.
E10	.	A	.	.	.	.	.	.	T	.	.	.	A	.	.	A	T	.	.	.	.	.	.	.	A	.
E11	.	.	.	.	1^c^	.	.	.	T	.	.	.	A	.	.	A	T	.	.	.	.	.	.	.	A	.

All sequences are compared to the reference haplotype H1. Numbers ‘0/1’ in the sequences indicate absence/presence of four length polymorphisms, and ‘2’ presence of an inversion, whereby superscripts identify corresponding character states. Note that poly-A or poly-T stretches were excluded from analyses.

a : AAGC;

b : AATC;

c :TTTTTATTA;

d :AATTTTA;

e : AT;

f : CCTCTTGATAGAACAAGAAAAA;

g :TTTTTCTTGTTCTATCAAGAGG.

Haplotype diversity and within-population diversity declined under anthropogenic influence during the course of cultivation (Table 3). Wild populations contained all haplotypes, whereas cultivated populations only had three haplotypes (A, B and E). Moreover, in wild *S. ningpoensis*, eleven out of fourteen populations (78.6%) were polymorphic for haplotypes. In contrast, every cultivated population was fixed for a single haplotype. Wild populations had much higher estimates of haplotype diversity (*h*_T_ = 0.919), the within-population component of diversity (*h*_S_ = 0.444) and nucleotide diversity (π_T_ = 0.00301) than do cultivated populations (*h*_T_ = 0.399 *h*_S_ = 0, π_T_ = 0.00076). In wild *S. ningpoensis*, haplotype diversity varied: populations LSW, TM2W had the highest haplotype diversity whereas populations TWW, PTW, THW from Zhejiang were each fixed for a single haplotype (Figure 1C). An AMOVA indicated that 41.02% of the total genetic variation occurred between cultivated and wild groups (Table 4), which is consistent with the high observed genetic differentiation (F*_ST_* = 0.71988) between cultivated and wild groups. And in cultivated *S. ningpoensis* all genetic variation was among populations, but in the wild populations, more variation occurred within populations (58.54%) than among wild populations (41.46%).

**Table 3:** Comparisons of genetic diversity and genetic structure between wild and cultivated *Scrophularia ningpoensis* populations based on chloroplast DNA sequences.

Parameter	wild	cultivated
Number of haplotype	21	3
haplotype diversity (*h*_T_)	0.919	0.399
Within-population diversity(*h*_S_)	0.444	0
Nucleotide diversity(π_T_),	0.00301	0.00076

**Table 4:** Hierarchical analysis of molecular variance for 28 populations of *Scrophularia ningpoensis* based on chloroplast DNA sequences.

Regional grouping of populations	Source of variation	d.f.	Sum of squares	Variance components	Percentage of variation	F-statistics(P)
Cultivated	Among populations	12	79.731	0.50613	100	*F*_ST_ = 1(<0.001)
	Within populations	158	0	0	0	
Wild	Among populations	13	156.171	0.79368	41.46	*F*_ST_ = 0.41462(<0.001)
	Within populations	179	200.576	1.12053	58.54	
Cultivated vs. wild	Among groups	1	167.858	0.87163	41.02	*F*_CT_ = 0.41023(<0.001)
	Among populations within groups	25	235.902	0.65791	30.96	*F*_SC_ = 0.52503(<0.001)
	Within populations	337	200.576	0.59518	28.01	*F*_ST_ = 0.71988(<0.001)

*F*_ST_: genetic differences among populations; *F*_CT_: genetic differences among groups defined a priori; *F*_SC_: genetic differences among population within groups.

Maximum parsimony analysis of these 21 haplotypes, with *S. buergeriana*, *S. spicata* and *S. dentata* as the outgroup, resulted in 501 most parsimonious trees with a length of 87, a consistency index (CI) of 0.828, and a retention index (RI) of 0.583. Maximum likelihood optimization resulted in a final optimization likelihood of −2020.22, with the alpha parameter being 0.020000. The best-scoring ML tree had a length of 0.073087. The Maximum likelihood tree with statistical support indicated above branches was presented in Figure 1A, haplotypes of A, B, C, C1, C2, C3, D, D1 and D2 were monophyletic but with very low bootstrap support. The MP/BI/ML analyses yield mostly congruent topologies. Haplotypes (A, B, E) which cultivated populations harbored occur in different clades. The cpDNA haplotype network for the 27 populations of *S. ningpoensisis* is shown in Figure 1B. The rooted TCS network (Figure 1B) showed a similar structure to the phylogenetic tree (Figure 1A), which grouped 21 haplotypes into two distinct clades. The outgroup *S. buergeriana* is linked with the center of the whole network by two missing haplotypes. Two most common haplotypes A and B are on tip position linked with haplotype C and D to form one clade, while the majority of rare haplotypes forms a “star” shape dominated by haplotype E. The geographic distribution and haplotype frequencies within populations are shown in Figure 1C. The most frequent haplotypes (A, B and E) were shared by both wild and cultivated groups, while other remaining haplotypes were found only in wild populations (Figure 1C). The most common and widest geographically distributed haplotype in cultivated populations was A which was unique to the wild population LSW located in Lenshui County in northeastern Jiangxi Province. In wild populations, there was no clear geographical pattern of within-population diversity (Figure 1C). Moreover, U test (Nst/Gst) indicated no phylogeographic structure in wild *S. ningpoensis* (N_ST_ = 0.446, G_ST_ = 0.534, U = −1.57, P>0.05).

### AFLP data

After exclusion of ambiguous or irreproducible fragments, 289 fragments were consistently scored for 24 populations (306 individuals) of which 261 fragments (90.31%) were polymorphic. The test for reproducibility yielded a mean error rate of 1.7% and confirmed the reproducibility of the AFLP fragments. Wild populations had much higher estimates of genetic diversity (*h* = 0.0791–0.1614) than did cultivated populations (*h* = 0.0076–0.0875) (Table 5). In wild *S. ningpoensis*, the percentage of polymorphic fragments varied from 22.15% in population TWW to 50.87% in population LAW; in cultivated *S. ningpoensis*, the percentage of polymorphic fragments varied from 3.11% in population SCC to 27.68% in population NSC (Table 5).

**Table 5:** Genetic diversity in 24 populations of *S. ningpoensis* based on AFLP.

	N	*N*_A_	*N*_E_	*h*	*H*	*F*_P_	*PPF*	*D*_W_	G_ST_	*N*_M_
**Cultivated**										
GZC	15	1.0796	1.0253	0.0177	0.0295	23	7.96	13.26		
JFC	13	1.2491	1.1515	0.0875	0.1307	72	24.91	8.38		
SCC	19	1.0311	1.0114	0.0076	0.0123	9	3.11	8.54		
XJC	10	1.0484	1.0236	0.0149	0.0232	14	4.84	8.68		
PAC	16	1.0657	1.0226	0.0150	0.0247	19	6.57	14.70		
YLC	10	1.0796	1.0326	0.0215	0.0345	23	7.96	4.80		
ZPC	10	1.1211	1.0597	0.0371	0.0576	35	12.11	7.72		
KKC	8	1.1280	1.0821	0.0467	0.0694	37	12.80	3.57		
LOC	10	1.1349	1.0458	0.0324	0.0537	39	13.49	5.79		
NSC	10	1.2768	1.1427	0.0874	0.1346	80	27.68	7.74		
**Wild**										
HNW	15	1.3322	1.1797	0.1083	0.1649	96	33.22	21.92		
JGW	15	1.4256	1.2273	0.1369	0.2088	123	42.56	24.79		
JHW	22	1.5052	1.2271	0.1405	0.2195	146	50.52	14.90		
JMW	13	1.3668	1.1982	0.1197	0.1825	106	36.68	20.05		
LAW	14	1.5087	1.2677	0.1614	0.2469	147	50.87	12.51		
LSW	15	1.4394	1.2559	0.1503	0.2260	127	43.94	10.81		
MTW	15	1.4221	1.2135	0.1291	0.1985	122	42.21	22.32		
PTW	9	1.2422	1.1340	0.0812	0.1235	70	24.22	5.40		
THW	14	1.4498	1.1980	0.1233	0.1943	130	44.98	9.77		
TNW	14	1.4429	1.2196	0.1345	0.2078	128	44.29	9.33		
TTW	10	1.4118	1.2271	0.1374	0.2092	119	41.18	6.87		
TM1W	10	1.2318	1.1402	0.0821	0.1231	67	23.18	6.33		
TM2W	10	1.3702	1.1929	0.1175	0.1806	107	37.02	5.39		
TWW	9	1.2215	1.1331	0.0791	0.1187	64	22.15	5.04		
Cultivated	121	1.654	1.2591	0.1554	0.2422	189	65.4		0.7568	0.1607
Wild	185	1.8893	1.3385	0.2171	0.3466	257	88.93		0.4352	0.6490
Species	306	1.9031	1.3551	0.2202	0.3481	261	90.31		0.6111	0.3181

***N*** **_A_:** Observed number of alleles; ***N*** **_E_:** Effective number of alleles [Kimura and Crow (1964)]; ***h*** **:** Nei's (1973) gene diversity; ***H*** **:** Shannon's Information index [Lewontin (1972)]; ***N*** **_M_:** estimate of gene flow from *G*_ST_ or *G*_CS_. E.g., *N*_M_ = 0.5(1−*G*_ST_)/*G*_ST_; **N:** the number of individuals in population; ***F*** **_P_:** the number of polymorphic fragments; ***PPF*** **:** the percentage of polymorphic fragments; ***D*** **_W_:** the frequency down-weighed marker value.

The variation between cultivated and wild groups accounted for 19.62% of the overall genetic variation (Table 6). In the cultivated group, most variation (76.97%) existed in among-population with only 23.03% due to the within-population (Table 6). But the variation distribution in the wild group was opposite that most variation (60.40%) existed in within-population with only 39.60% due to the among-population (Table 6).

**Table 6:** Hierarchical analysis of molecular variance for 24 populations of *S. ningpoensis* based on AFLP.

Regional grouping of populations	Source of variation	d.f.	Sum of squares	Variance components	Percentage of variation	*F*-statistics
Cultivated	Among populations	9	2251.916	18.72650	76.97	*F*_ST_ = 0.76974
	Within populations	111	666.615	5.60181	23.03	
Wild	Among populations	13	2451.695	12.85266	39.60	*F*_ST_ = 0.39605
	Within populations	171	3351.516	19.59951	60.40	
Cultivated vs. wild	Among groups	1	1299.331	7.09433	19.62	*F*_CT_ = 0.19616
	Among populations within groups	22	4703.610	15.21535	42.07	*F*_SC_ = 0.52339
	Within populations	282	4018.131	13.85563	38.31	*F*_ST_ = 0.61688

*F*_ST_: genetic differences among populations; *F*_CT_: genetic differences among groups defined a priori; *F*_SC_: genetic differences among population within groups.

**Figure 2:**
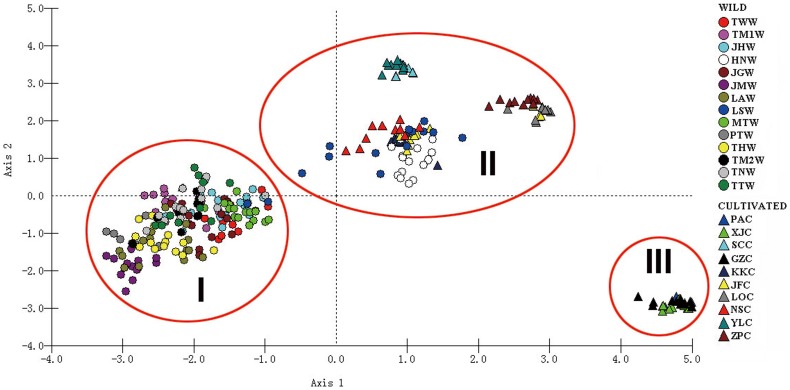
Principal coordinate analysis (PCoA) of 306 individuals from 24 populations of *S. ningpoensis* based on the Euclidean distance generated from AFLP data. To simplify comparison, cultivated *S. ningpoensis* are indicated by triangles, while wild *S. ningpoensis* are indicated by circles.

**Figure 3:**
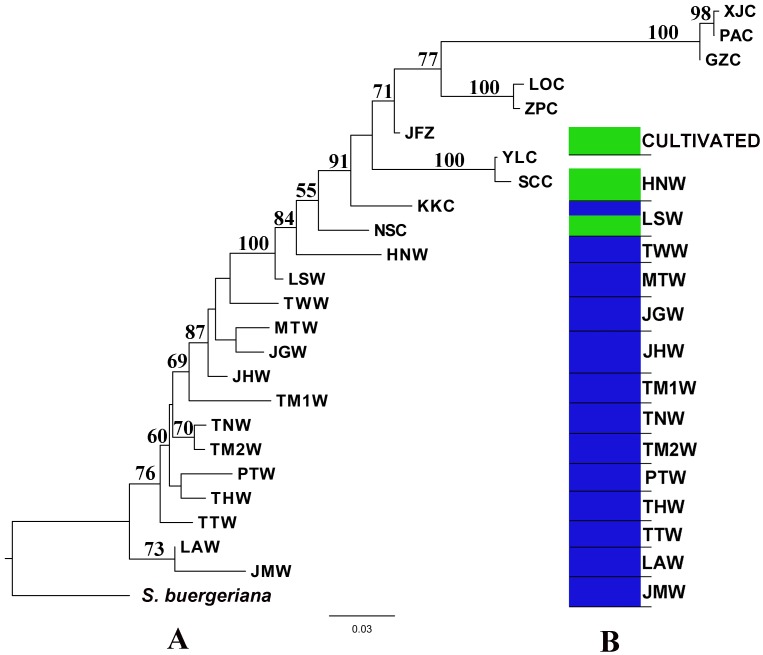
Neighbor-Joining and STRUCTURE analyses of the AFLP data. (A) Neighbor-Joining analysis of the AFLP data for all individuals of *S.ningpoensis* based on Nei's (1979) genetic distances with *S. buergeriana* as the outgroup. (B) Result of the model from clustering (K = 2) of all wild individuals and 10 cultivated individuals of *S. ningpoensis* using STRUCTURE based on the AFLP data set.

**Figure 4:**
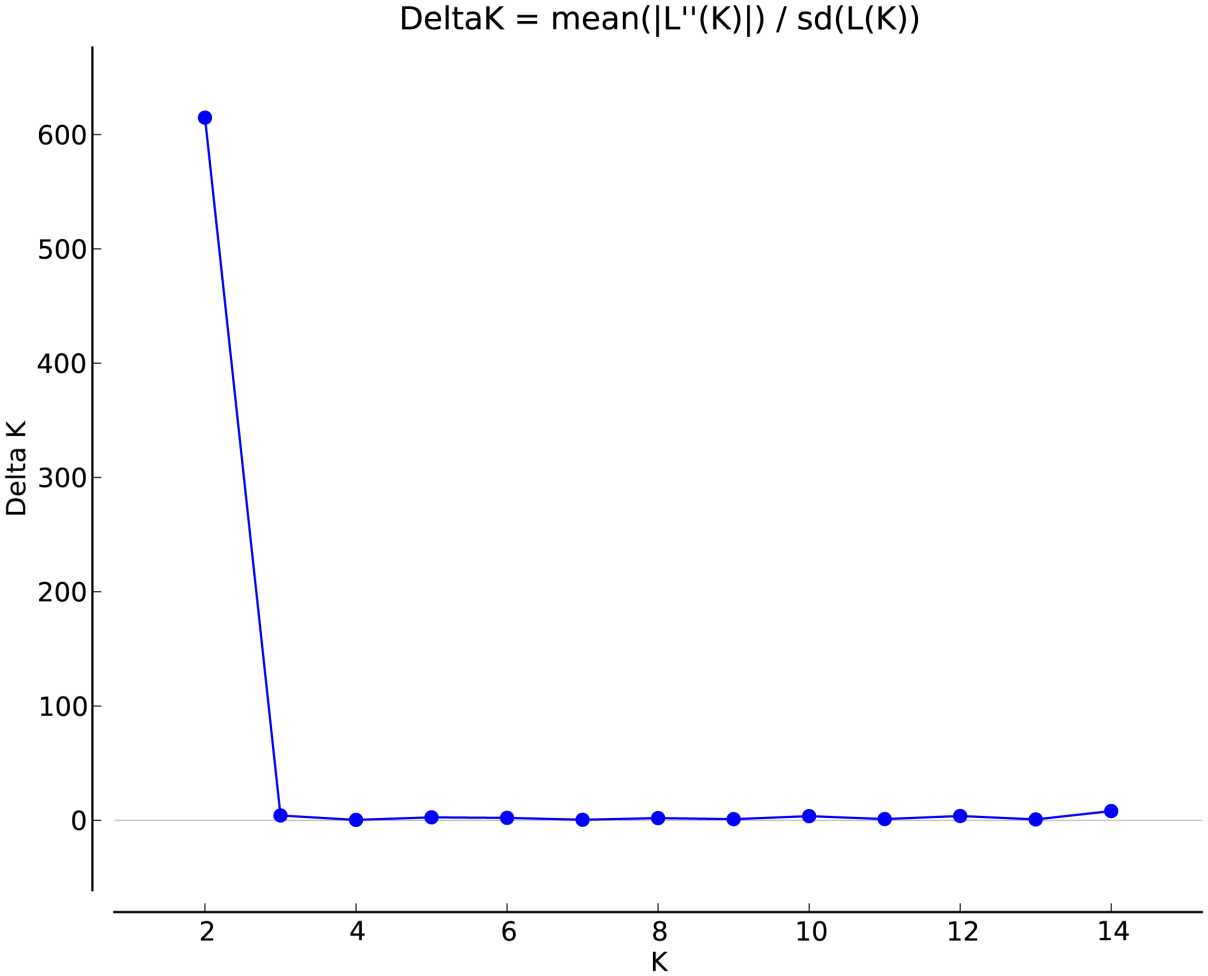
The estimated mean logarithmic likelihood of K values ranging from 2 to 14 with 10 replicates for each K calculated using the R-script Structure–sum.

Principal coordinate analysis also showed a clear differentiation between cultivated and wild *S. ningpoensis*. All populations were grouped into three main clusters (Figure 2): cluster I included most wild populations; cluster II included most cultivated populations and two wild populations LSW & HNW; and cluster III included three cultivated populations: PAC, XJC and GZC. Populations PAC and XJC were from Zhejiang province. While GZC is in Fujian Province, local farmers indicated that it was directly introduced from Zhejiang cultivated populations. Genetic admixture analysis performed by STRUCTURE divided genetic variation into two gene pools (k = 2, Figure 3B): (1) Cultivated individuals, wild population HNW and part of wild population LSW; (2) Other wild populations. In STUCTURE analysis K values ranged from 2 to 14 and the one with the highest likelihood (k = 2) was chosen (Figure 4).

The Neighbor-Joining analysis identified two wild populations (HNW & LSW) that had a close relationship with cultivated *S. ningpoensis*. The wild population HNW was sister to cultivated populations with a bootstrap value of 84%; LSW clustered with HNW and all cultivated populations supported by 100% bootstrap value (Figure 3A). Although three cultivated populations, PAC, XJC and GZC, grouped together and are distinct from other *S. ningpoensis* in PCoA analysis, in the NJ tree they located in one clade with hundred percent support and were associated with other cultivated populations.

## Discussion

### Change of genetic diversity under domestication

For many crop species the domestication process has left a signature in the pattern of genetic diversity as a result of founder effects, genetic bottlenecks and artificial selection [76]. Many crops show evidence of genome-wide reductions in variation [77], [78], [79]. In our study, the pattern of genetic diversity between cultivated and wild populations of *Scrophularia ningpoensis* is consistent with other domesticated crop species, even though the domestication history of *S. ningpoensis* is only one thousand years. Out of twenty-one cpDNA haplotypes in the species, only three haplotypes were detected in cultivated populations. Moreover, cultivated populations had little within-population genetic polymorphisms (Table 3). The cpDNA nucleotide diversity (*π*_T_) and haplotype diversity (*h*_T_) of cultivated populations were much less than that of the wild group (Table 3). AFLP data also support these patterns (Table 5). These results are consistence with many crop-relative systems of domestication [80], [81] although the degree of decline in diversity of *S. ningpoensis* with such a short history of domestication is surprising. This medicinal plant has lost genetic diversity as a result of domestication which clearly shows that a species can have large genetic alterations even before there are significant changes in morphology, phenology or breeding system. Other species native to China, such as *Corydalis yanhusuo* (traditional Chinese medicine) [16], *Metasequoia glyptostroboides* [82], *Malus pumila* [83] and *Zizania latifolia* [84] also revealed the same genetic pattern.

These large changes in genetic diversity are most likely the result of both founder effects and selection for medicinal quality, and importantly, the clonal mode of reproduction. The extent of the loss of genetic diversity depends in part on population size during domestication and time over which domestication occurred [77]. At the beginning of cultivation, *S. ningpoensis* was gathered from the wild. Most likely a limited number of individuals from the wild formed the founding populations of cultivated *S. ningpoensis*, the founders containing only a sample of the genetic diversity of wild populations. Although the domestication history of *S. ningpoensis* is short, under cultivation it is propagated vegetatively from rhizome and flowers are removed. These practices restrict gene flow between wild and cultivated *S. ningpoensis* populations and among individuals within cultivated populations. Moreover, farmers select large and healthy rhizomes to establish new populations. Such selection coupled with clonally propagation could quickly fix alleles carried by selected individuals. The increased *F*_ST_ values (Table 4, Table 6) for cultivated populations (cpDNA: *F*_ST_ = 1; AFLP: *F*_ST_ = 0.76974) relative to wild populations (cpDNA: *F*_ST_ = 0.41462; AFLP: *F*_ST_ = 0.39605) reflect the different modes of reproduction in cultivated (clonally propagated) and wild (sexually propagated) populations.

### Origin of cultivated *Scrophularia ningpoensis*

Locating the geographical site of domestication and assessing the demographic consequences of wild-domesticated species evolution can help to design strategies of use and management of genetic resources [85]. In the current study, three cpDNA haplotypes (A, B and E) found within cultivated populations are scattered on the haplotype network and shared with different wild population gene pools. But the most cultivated populations harbored haplotype A, which is only found in the wild population LSW. Two other haplotypes B & E, distributed in two cultivated populations (YLC and SCC) and one cultivated population (NSC) respectively, are shared with three and four different wild populations respectively. It is interesting that wild populations harboring haplotype B or E didn't have haplotype A. This cpDNA pattern indicates that the wild population LSW in Jiangxi Province contributed substantially to the origin of cultivated *S. ningpoensis*, while other six wild populations (JGW, LAW, JHW, TNW, TM2W, PTW) located in geographically separated places were only involved in origin of three cultivated populations. Moreover, the wild population JGW harboring both haplotype B and E is also in Jiangxi Province as the wild population LSW. Thus, the Jiangxi region is a critical site for the origin of cultivated *S. ningpoensis*.

However, results of AFLP analysis suggested that the origin of cultivated populations is more restricted to only two wild populations, HNW and LSW. In the neighbor-Joining analysis (Figure 3), HNW and LSW clustered with other cultivated populations by 100% bootstrap value and the STRUCTURE analysis was consistent, placing the two wild populations, HNW and LSW, into the same gene pool as the cultivated individuals. In principal coordinate analysis (Figure 2), wild population HNW and LSW grouped with majority of cultivated populations in one cluster. Thus, combined with cpDNA data, we infer that wild populations of the Jiangxi region may have played a major role in the origin of cultivated *S. ningpoensis*. Although three cultivated populations, PAC, XJC and GZC, grouped to another cluster in PCoA analysis (Figure 2), they still located in the cultivated clade identified in the Neighbor-Joining analysis (Figure 3A) and were assigned to the same gene pool as the other cultivated *S. ningpoensis* (Figure 3B). The wild population LSW located in eastern Jiangxi and HNW population is on the border between Hunan and Jiangxi, so AFLP data strongly supported that Jiangxi area of Eastern China is the geographic origin of cultivated *S. ningpoensis*.

Considering the discordance between chloroplast and nuclear markers,the origin of cultivated *S. ningpoensis* must be interpreted with caution. Firstly, patterns of cpDNA indicated broader geographic origin of cultivated *S. ningpoensis* compared with AFLP markers, which can be explained by the characteristics of chloroplast genome evolution. CpDNA evolves slowly and is uniparentally inherited in plants, so it usually only represents the maternal genome and often has low levels of variation making cpDNA less informative for intraspecific studies [86]. Nuclear genomic AFLP markers provided much higher resolution both because of rapid evolution and many independent markers, thus providing a more nuanced interpretation of the origin of cultivated *S. ningpoensis*. Revealed by AFLP markers, cultivated populations originated from Jiangxi area where wild populations were involved in the event of origin. Secondly, if AFLP data has revealed the more resolution, these two wild populations which were involved in the origin of cultivation, HNW & LSW, should harbor all cpDNA haplotypes (A, B and E) found in cultivated populations. But the wild population HNW didn't share any cpDNA haplotype with cultivated populations; only the native population LSW harbored haplotype A. These data suggest that this entire region of China may have served as the source of medicinal *S. ningpoensis* rather than a specific population. The ancestors of cultivated populations YLC, SCC and NSC were probably introduced from wild population similar to the extant HNW; harvesting and cultivation may, by chance, collected all individuals which had the same cpDNA haplotypes with cultivated populations. In addition, the nearest wild population to HNW is JGW, which shared two cpDNA haplotypes, B&E, with cultivated populations. But the very high value of Dw in HNW (21.92) and JGW (24.79) suggests a long-term history of isolation [74]. The long term use, and most likely, trading of rhizomes of *S. ningpoensis* lead to a complicated domestication history for this species.

### Crop improvement and sustainable cultivation of *S. ningpoensis*

The therapeutic efficacy of a medicinal plant often varies by geographical location among the populations of a species [22]. Cultivated *S. ningpoensis* from Zhejiang Province is recognized to have the best medicinal quality [87]. Our previous study on HPLC fingerprints of *S. ningpoensis* has also revealed that materials cultivated in Zhejiang Province produce the highest content of bioactive compounds compared to other cultivated populations [45]. In this study, analysis of AFLP markers group cultivated populations from Zhejiang into one cluster which is divergent from other cultivated populations and other wild populations (Figure 2). Moreover, cultivated populations from Zhejiang gather in one clade associated with other cultivated populations in NJ tree (Figure 3A). This evidence strongly supports the genetic distinct nature of cultivated *S. ningpoensis* from Zhejiang (Figure 2). Under strong artificial selection, cultivated *S. ningpoensis* with high pharmaceutical quality has genetically diverged from wild populations. It is generally believed that different medical effects due to secondary compounds are caused by various environmental conditions and habitats where the medicinal plants are grown and harvested [88]. But our study combined with previous phytochemical analysis indicates that under artificial selection, cultivated *S. ningpoensis* from Zhejiang has become genetically differentiated from other cultivated populations and chemical diversity may also be influenced by genetic factors. Thus, this result can help design strategies of crop improvement that consider both genetic and ecological factors for the cultivation of medicinal plants.

Genetic diversity underlies the plasticity of many secondary metabolites [89] and results in phytochemical diversity on which the pharmaceutical quality of medicinal plants relies [90]. Study on *S. ningpoensis* by HPLC and ISSR fingerprinting have already revealed a strong association between chemical and genetic variation of *S. ningpoensis* [45] suggesting that higher genetic diversity can account for higher phytochemical diversity that is critical for the pharmaceutical quality. This study that compares cultivated populations to the range of extant wild populations indicates that wild *S. nignpoensis* has much higher genetic diversity than does the cultivated plant (Figure 1C, Table 5). These wild populations represent an infrequently used source of genetic diversity that is conspecific with the cultivated gene pool. Cultivated and wild plants generally do not exhibit reproductive isolation [79]. Hence, wild resources can be utilized to improve the cultivated *S. ningpoensis* by introducing genetic diversity by sexual reproduction and then selection for desired traits.

The results of this study can be used to select the most diverse natural populations for such a breeding program. Using the AFLP data, the values of Nei's [67] gene diversity (*h*) of most wild *S. ningpoensis* were higher than 0.1 (Table 5), except for PTW, TWW and TM1W. For chloroplast data, three wild populations, TM2W, LSW and JHW, harbored more cpDNA haplotypes than others (Figure 1C). Thus, TM2W, LSW and JHW populations have high genetic diversity for both the cpDNA and nuclear genomes. These native populations have the greatest reservoir of genetic diversity and could be used for further improvement of cultivated *S. ningpoensis* to ensure that it provides an adequate supply of the medicinal plant and the quality of the plant is enhanced. Furthermore, the utilization of wild populations with appropriate guidelines for collection and for regulating management could be an efficient way to find, conserve and deploy desirable agronomic traits which would be beneficial for Radix Scrophulariae cultivation.
